# Using Quantitative Systesm Pharmacology to optimize amyloid‐tau combination clinical trials on Alzheimer's Disease

**DOI:** 10.1002/alz70859_099977

**Published:** 2025-12-25

**Authors:** Hugo Geerts, Shaina M Short

**Affiliations:** ^1^ Certara Predictive Technologies, Berwyn, PA USA; ^2^ Certara Predictive Technologies, Park City, UT USA

## Abstract

**Background:**

With the recent approval of amyloid antibodies for the treatment of Alzheimer’s Disease, emphasis is now shifting to combination therapies with disease‐modifying therapeutics such as tau, against the background of small molecules affecting neurotransmitter pathways such as cholinergics, benzodiazepines and antidepressants. Key challenges include timing, dosing and pharmacodynamic interactions with regard to biomarkers and functional clinical outcomes

**Method:**

We used a computational neuroscience model of a cortical microcircuit, previously calibrated to ADAS‐Cog with implementation of the effect of amyloid monomers on NMDA and a7nACHR, amyloid oligomers on AMPA and K+ conductance. Misfolded tau proteins affected both K+ channels and Na+ channels, especially in the Axon Initial Segment.

**Result:**

Model outcomes suggest a complex interaction between disease state, amyloid and tau pathology baseline. Pathological tau leads to a greater cognitive improvement in AD vs MCI but the improvement in MCI patients increases with higher baseline amyloid load, while this is much less affected in AD disease state. With regard to APOE genotype, reduction in pathological tau improves non APOE4 carriers more for low baseline amyloid load as compared to APOE4 carriers only in AD pathology. For higher baseline Amyloid, pathological tau reduction leads to a greater improvement for intermediate baseline tau load (1.3<SUVR<1.9) for all APOE carriers.

**Conclusion:**

The simulations suggest a complex interaction between disease state, amyloid and tau baseline and to a lesser extent APOE genotype for the effect of pathological tau reduction on functional clinical scales. Failure to take this interaction into account might lead to reduced clinical signals in tau therapeutics drug development. Importantly, this model can also be used for appropriate combination therapy of amyloid and tau therapeutics against the background of comedications in AD patients.

